# Listen to the Voices of Unwed Teenage Mothers in Malaysian Shelter Homes: An Explorative Study

**DOI:** 10.5539/gjhs.v5n5p20

**Published:** 2013-05-21

**Authors:** Nor Jana Saim, Mona Dufåker, Malin Eriksson, Mehdi Ghazinour

**Affiliations:** 1 Department of Social Work, Umeå University, Umeå, Sweden; 2 Department of Public Health and Clinical Medicine, Umeå University, Umeå, Sweden; 3 School of Psychology and Human Development, Universiti Kebangsaan Malaysia, Kebangsaan, Malaysia

**Keywords:** unwed teenage mothers, shelter home, residential care, social work, Malaysia

## Abstract

This qualitative research aims to explore the daily life experiences of Malaysian unwed teenage mothers in shelter homes. The research is based on the thematic analysis of interviews with seventeen respondents aged from 12 to 18 years. Eight sub-themes described the experience of the unwed teenage mothers in the shelter home and led to three overall themes: rules and regulations, relationship with the staff and relationship with the other girls at the shelter home. The findings indicated that the shelter homes involved were not fulfilling the standard of the Malaysian national laws and United Nations Convention on the Rights of the Child. We strongly suggest that the authorities provide a clear guideline concerning the implementation of Malaysian national laws and United Nations Convention on the Rights of the Child.

## 1. Introduction

In Malaysia, the number of teenagers represent about 20 percent of the 28 million population (Department of Statistics Malaysia, 2011). Several studies have been conducted concerning teenagers, especially about education, self-esteem, stress, family relationship, substance abuse and resilience (Hashim & Borhan, 2007). One issue of concern among teenagers is pregnancy out of wedlock (Fatimah, 2009; Jamaludin, 2010; Mohamed, 1993).

It is reported that, in 2009, there were 2,049 cases of statutory rape and the number of cases increased to 2,419 in 2011 (Abu Bakar, Abdullah, & Maslih, 2012). Meanwhile, the mainstream newspapers reported that there were 70.430 illegitimate children registered from 1999 to 2003, and, from 2000 to 2008, the number had increased almost twofold (Mat Zaib, Sidek, Wahid, & Sheiakha, 2008). Research done in Sibu, Malaysia, found that teenage pregnancy constitutes about 12% of the total fertility rate and among one third are due to pregnancies of unwed mothers (Kawi, 2011).

The increasing number has attracted the attention of the Malaysian government to deal with the issue inasmuch as pregnancy could jeopardize teenage life (Omar et al., 2010) through dropping out from school, violence or delinquency (Barnet et al., 2009). Studies have shown that pregnancy out of wedlock is the main factor for infanticide and the abandonment of babies (Badiah & Mohd Jamil, 2006; Nasir, 2010).

Our aim is to listen to the voices of unwed teenage mothers living in shelter homes concerning their experiences in order to shed light on certain aspects of their situation. In the following sections we start with an overview of how the Malaysian legislation deals with unwed teenage mothers. Subsequently, we report on the life experiences of unwed teenage mothers. The international mandate, the Convention on the Rights of the Child, will be our framework in order to problematize and reflect on the interventions provided by the Malaysian authorities for unwed teenage mothers.

### 1.1 The Malaysian Legislation and the Unwed Teenage Mother

One of the options to deal with pregnancy out of wedlock other than marriage and abortion, is by sending the unwed mother to a maternity home (Rains, 2009). In Malaysia, the shelter home services for unwed teenage mothers were established fifty years ago. The admission normally needs a request either from the unwed teenage mother or her parents/family. If the girls are under 18 years, the admission is due to a court order. There are three types of shelter home administration – governmental, semi-governmental and non-governmental organization, which are the most common. The governmental and semi-governmental shelter homes are free of charge, while, normally, the charge for the non-governmental shelter homes ranges from RM300 (USD96) to RM2.500 (USD801) depending on the services provided. In 2012, there were in total five governmental shelter homes in Malaysia, one located in the centre (Kuala Lumpur), one in the north, one in the south and two in East Malaysia (Malaysian Borneo), thus covering the whole country.

The shelter homes are all under the Malaysian Care Centre Act (2006). As the unwed teenage mothers are considered as being exposed to sexual abuse and in urgent need of protection, according to the Malaysian Child Act (2006), the shelter homes should accordingly serve as residential correctional facilities to prevent recidivism of pregnancy out of wedlock by giving protection, supervision, rehabilitation and training (Care Centre Act, 2006; Dar Assaadah, 2008). The person in charge of the shelter home shall bring the girl with a full report of the circumstances before a Protector, usually a Social Welfare Officer, within twenty-four hours of her admission into the shelter home. The protector shall then immediately inform the Court for Children of such an admission as well as her discharge (Child Act, 2006).

The minimum period for residing in a shelter home is two weeks, and the maximum duration is almost two years, depending on the girls’ needs. In general, the shelter homes offer various activities from vocational classes (e.g. tailoring classes and handicrafts), outdoor and religious activities, job placement as well as voluntary work. The vocational and religious classes are usually conducted by volunteers. Some of the shelter homes offer vocational classes with a certificate from a private college. The rules and regulations vary from one shelter home to another. In a governmental shelter home, the social workers are in charge of the girls, while in the NGO shelter homes, a warden or the owner are in charge of them and are normally assisted by administrative staff. Generally the shelter homes accommodate security staff, cooks and drivers. If there is no cook in the shelter home the girls need to prepare the food themselves. Usually the shelter homes also offer consultation concerning adoption if a girl's family decides to give the baby up for adoption.

### 1.2 Literature Review on Unwed Teenage Mothers

The issue of unwed teenage mothers is often studied from different perspectives, such as risk factors (Jamaludin, 2010; Saim, 2004), maternal complications and reproduction (Md Zain, 2002; Suryoputra, Ford, & Zahroh, 2006), the rights of the illegitimate children (Mohd Awal, 2009; Mohd Wahie, 2004), prohibition according to religious and societal norms and shame to the family (Azizi, 2010; Mohamed, 1993). In Asian culture, being pregnant out of wedlock is associated with misbehaviour, being a pervert and significantly bringing shame and dishonour to the family (Chhabra, 1992; Kenneth & Kasper, 1992; Triwulan, 2009; Wang, 1997) or seen as an unfortunate event (Kurup, Viegas, Singh, & Ratnam, 1989). Due to the shame of being pregnant out of wedlock the girl is either encouraged to marry the father of the baby (usually her boyfriend), undergo an abortion, give up the baby for adoption or is sent to a shelter home in order to hide the pregnancy (Rains, 2009).

A few empirical studies exist on Malaysian unwed teenage mothers in shelter homes. One study focused on the social interaction between the staff and the girls (Abdullah, 2010), one on the romantic relationship (Abd Ghadur & Abdul Kadir, 2009), and others on the effectiveness of the programmes offered in the shelter homes (Azizi & Abd Wahab, 2008; Azizi, Abdul Rahman, Hamdan, & Boon, 2010). Emond (2003) emphasized that the perspective about the day-to-day experiences of young people who reside in residential care receive less attention although studies about residential care has been conducted for many years.

Abdullah (2010) found that girls who were admitted in a shelter home due to delinquent behaviour developed pseudo-family relationships among themselves. They shared their feelings as well as their belongings, such as toiletries and food. At the same time, the girls believed that the programmes provided by shelter homes were less effective due to unqualified counsellors, uninteresting moral classes and outdated facilities (Azizi, 2010). Additionally, another survey study in two different shelter homes indicated that the religious classes were more effective compared to the vocational, co-curriculum and counselling activities (Azizi & Abd Wahab, 2008). Furthermore, a study conducted in the United States by Marshall and Baffour (2011) on the experiences of young people residing in a long-term care facility found that their concern was about social and family disengagement, non-stimulating activities and the relationship between staff and residents.

### 1.3 Convention on the Right of the Child

An accredited United Nations [UN] mandate, the Convention on the Right of the Child (United Nations [UN] General Assembly, 1989), functions as our theoretical framework since the Malaysian government has ratified the Convention (Convention on the Rights of the Child, 1989). According to Article 2, every child has the same rights and equal value regardless of race, religion, culture, language, thinking or say. This article emphasizes that one should not judge or treat any child with discrimination. The child deserves to be treated with respect. The Convention also emphasizes the provisions regarding the rights for children's opinions to be heard. Article 12 states that the child has a right to express her/his opinions in accordance with her/his age and maturity as well as encourage the child's involvement in the decision-making. It is strongly recommended that the authority should listen, respect and take the children's opinions into consideration. Further, Articles 13 and 14 indicate that freedom of thought and expression could vary, either orally, in writing or in print, in the form of art, depending on the child's choice as long as it is not damaging to her/him or others.

Referring to Article 3, the best interest of the child must be a priority in all actions concerning children. The article also pinpoints the responsibilities of the authority to ensure that the institutions, services and facilities responsible for the care or protection of children shall conform to the standards established by competent authorities, particularly in the areas of safety, health, in the number and suitability of their staff, as well as competence. Article 6 and 27 in the Convention indicate that the authority shall ensure, to the maximum extent possible, the survival and development of a child to a standard of living in regards to her/his physical, mental, spiritual, moral, psychological and social development. Article 4 emphasizes that the authority should be obliged to take all necessary steps to ensure that the children's rights are respected protected and fulfilled.

The Convention highlights in Article 19 the importance of the authority in ensuring the well-being of children from all forms of physical or mental violence, injury or abuse, neglect or negligent treatment, maltreatment or exploitation and sexual abuse. The same article also states that the authority is responsible to provide social programmes that could offer necessary support, prevention and identification, reporting and referral, investigation and follow-up.

The provision in Article 20 states that a child who cannot be looked after by her/his family has a right to special care and must be looked after properly, by people who respect his/her ethnic group, religion, culture and language. Article 25 emphasizes that the authority should follow-up the care living arrangement for the child to ensure it is the most appropriate for the best interest of the child. It is affirmed in Article 9 and 37 that the care should also respect the right of the child to maintain a personal relationship and direct contact with parents on a regular basis, unless it is against the best interests of the child (UN General Assembly, 1989). Article 39 noted that the care for children should concern physical and psychological recovery and social reintegration in an environment that fosters the health, self-respect and dignity of the child.

## 2. Methodology

### 2.1 Sample

The first author contacted via email or telephones all the shelter homes for unwed mothers located in Kuala Lumpur and Selangor, Malaysia. The catchment area was selected due to the location and accessibility. The shelter homes were informed about the aim of the study and the research methods. Thirteen out of the twenty-two shelter homes did not give any response to our proposal. Nine agreed to participate but five shelter homes that agreed to take part did not have any unmarried teenage mothers placed in their facilities at that time. The other four shelter homes participated; one was a governmental shelter home and the other three were NGO shelter homes.

As the respondents were considered as a hard to reach population (Abrams, 2010; Earthy & Cronin, 2008), convenience and purposive sampling were used to select respondents for interviews (Marshall, 1996; Miles & Huberman, 1994). Purposive sampling also aims for maximal variation of the data (Patton, 2002). Sampling of respondents was done until saturation was reached, i.e. when no substantial new information with regards to the research questions were collected (Dahlgren, Emmelin, & Winkvist, 2007). In total 17 respondents were interviewed, of which twelve were residing in the governmental shelter home whilst five were residing in the NGO shelter homes. The inclusion criteria were: (a) have no severe psychiatric disorder or drug abuse and (b) no severe somatic illness. These inclusion criteria were communicated to the social workers at the shelter homes who invited respondents based on their knowledge of the current and former health status of the girls. The respondents ages ranged from 12 to 18 years due to the Malaysian legislation that considers the teenage period to be from 10 to 18 years (Child Act, 2006; Najmuddin, 2007).

### 2.2 Data Collection

The narrative interviews took an average of about one hour for each session. All interviews were conducted by the first author in Malaysian language. The interviews were held in an informal way in order to create a natural feeling and make it comfortable for the respondents to talk about their experiences (Elliot, 2005). The first author addressed herself as “kakak” which means “older sister” and is an informal form of address in Malaysian culture to be close to someone. A thematic interview guide was used that contained questions about experiences of internal (within the homes) and external (outside the homes) relations and support, and various views and experiences of staying in shelter homes. The time for the interviews was set according to the agreement between the first author and the respondents. Most of the interviews were conducted during the respondents’ free time. Some of the interviews were held during their scheduled activities, thus requiring the girl to be excused by her teacher or social worker. Most of the interviews were conducted in a private room at the shelter homes, while some took place in a car or in the garden if a private room was not available. All of the interviews were audio-recorded and transcribed.

### 2.3 Coding and Analysis

The interviews were transcribed, translated into English and coded by NVIVO version 9.0. As the respondent's views on the research topics were unfamiliar, we opted for thematic analysis (Braun & Clarke, 2006). The analysis was based on Braun and Clarke's (2006) recommendations and started by reading the transcriptions several times to become familiar with the data. The researchers had several discussions on the codes and the contents of the interviews during the process of generating, sub-themes and themes. The sub-themes and themes were reviewed and defined in order to confirm that it should reflect the explicit and implicit ideas expressed by the respondents.

### 2.4 Trustworthiness

Trustworthiness of a qualitative study is mainly judged on the level of transparency and reflections on how well the sampling, data collection and analysis were designed to address the research questions (Dahlgren et al., 2007). In this study, prolonged engagement in the research setting by the first author allowed us to get a comprehensive understanding of the shelter homes and the experiences of the unwed teenage mothers in these homes. Further, research triangulation was used by continuous negotiations and discussions among the research group about the preliminary findings. Since the research group represents different expertise and cultural understandings, this allowed us to understand and evaluate the findings from different perspectives. The first author is a native Malaysian researcher and social worker with experiences of working with vulnerable girls in Malaysia. The second and the third authors are Swedish researchers and social workers with experiences from the mental- and public health field. The last author is a Swedish-Iranian researcher and social worker with experiences of social work in multi-cultural settings.

### 2.5 Ethical Consideration

The ethical standards were fulfilled by the formal ethical approval of the Economic Planning Unit, Prime Minister's Department of Malaysia, and the Social Welfare Department of Malaysia, as well as from each shelter home involved. Prior to the interviews the respondents were informed about the study purpose, their role and rights, confidentiality, the data collection method and their voluntary participation. The respondents were also informed that they could withdraw from the study at any time. During the data collection, the first author was ready to provide help and support; if necessary.

## 3. RESULTS

The 17 respondents were in the ages of 12 to 18 years. One of the respondents was Indian, and the rest were Malays. Fourteen of the girls were still studying while three of them had terminated their schooling before they became pregnant. The thematic analysis resulted in three overall themes and eight sub-themes, describing the experiences of these unwed teenage mothers living in shelter homes. Quotations from the interviews are included to illustrate how the results are based in the data.

### 3.1 Rules and Regulations

#### 3.1.1 Restrictions Causes Social Limitations

Almost all of the respondents mentioned experiences of social limitations either directly from the rules and regulations imposed or when the rules reflected on the contact with their family. Most of the shelter homes had rules and regulations regarding this matter, for example, no family visit was allowed for the first month, only family and close relatives were allowed to visit once a month and one hour at a time. One of the shelter homes only allowed one phone call per week to their families while the others only allowed the girls to receive phone calls from family but not to make any of their own. Some of the families were reluctant to call the girls as the girls were perceived as wrongdoers by being pregnant out of wedlock: “I think I need more family support while staying here. But my mom rarely calls me. She just called me once since I came. My aunt also did the same.”

Some girls expressed that they saw their families as a source of information from the outside world and they were strengthened through their visits, letters and phone calls. Whilst some of the girls were craving for family support due to the restrictions set by the shelter homes. However, two of respondents did not complain about the social limitations as one of them had a family visit almost every week, while the other girl had been ‘rejected’ by her family since she was a young child and being in the shelter home made her feel better than being with her family.

#### 3.1.2 Unclear Rules Lead to Uncertainty

Almost all of the girls reflected that they were uncertain about the rules and regulations in the shelter homes. These respondents commonly mentioned that most of the information regarding the rules and regulations was explained by the other girls, who were senior in the shelter homes. The rules and regulations were sometimes not clearly explained or when they were not mentally ready to listen as they had just been placed in the shelter homes. Thus, they knew about the rules and regulations in brief but not in depth. They were aware of some aspects of the rules, but not others. This situation made them insecure concerning the expectations from the administration of the shelter homes:

*After shower we need to be downstairs [for dinner]. If we want to be upstairs [bedrooms] other than the time allowed we need to inform the social worker in charge. Otherwise we can’t do that. But I am not sure what the punishment is if I’m upstairs without informing the social worker. The social worker also told me but she told me a lot [rules]. I can’t remember what she told me*.

Some of the girls viewed certain rules and regulations in the shelter homes as absurd. Such rules as turning on a loudspeaker when making and receiving calls from their parents, no parental visit during hospitalization after giving birth, no family visit allowed if they broke the rules, and no stationery or books allowed in the bedroom. Two girls mentioned that the last was a problem as writing about their feelings was their main way of coping. Another respondent expressed her feelings about the banning of reading a newspaper in the shelter home she resided in: “She [the warden] banished one of the girls because she read a newspaper. We can’t read a newspaper like Harian Metro or entertainment magazine. We got a newspaper when she [the warden] threw her garbage. Then we took and read it. We are not stupid. We are not that stupid.”

#### 3.1.3 Regulated Domestic Tasks Promote Independence

All of the shelter homes had a day-to-day roster for the girls to do domestic chores. Two of the shelter homes had specific staff who worked in the kitchen as well as in the living area and the girls were scheduled to help them as their assistants. The other two shelter homes did not have a cook or housekeeper, so the girls needed to cook and clean the house as scheduled. There was a warden in charge to monitor their work from time to time.

Most of the girls perceived the household schedule as a way to teach them to be independent as most of the girls were inexperienced concerning domestic work. The girls shared their duties and helped each other:

*We just throw anything in the pot to cook [laughing]. There are 30 girls in the house. So we need to cooperate and ask around if anyone knows how to cook. Otherwise we just throw anything into the pot to cook. If it turns out to be good then we could do the same next time, but if it doesn’t then we need to modify here and there*.

In addition, all of the shelter homes provided religious classes while only two of them provided vocational classes and only one shelter home offered computer classes. In one shelter home, vocational classes were scheduled based on the free time of volunteers, while the other provided their regular staff. Two shelter homes had religious classes every day. There were strong tendencies that the respondents wanted to participate in all classes. Several girls said that they had too much free time without doing anything. Most of the girls talked about the positive impact of religious classes, compared to the other classes, as they felt relief:

*Thank God I stayed here. I didn’t know how to perform prayers, I didn’t know how to cook, but now thank God I know a lot of things. The staff have taught us. I didn’t know how to do the household chores, to plant a tree but I do now. They have taught us to do these things*.

### 3.2 Relationship with Staff

#### 3.2.1 Lack of Time and Trust Leads to Emotional Insecurity

The girls described that they felt emotionally insecure to share their feelings or problems. The majority of the respondents mentioned that they rarely shared their feelings with the social worker in charge or the staff who was appointed as their guardian. The girls said they hardly had one session with the social workers in charge even though that was among the main reasons the families sent them to the shelter homes. Several girls said that they were keen to share their feelings but no one was willing to listen. One of the girls thought that she could have counselling sessions and improve her behaviour; unfortunately the shelter home she resided in did not have that service. Some of them felt insecure as the social workers in charge glared at them or did not believe what they said or did nothing regarding their problems: “I kept losing my stuff; a pair of slippers, shirt, brush and such. But who am I going to talk to? I told the social worker but she didn’t believe me.”

Another girl shared her experience but the social worker who she confided in passed it on to others in the shelter home:

*I heard that the other social workers or maybe the nurse or maybe my social worker told them [the girls in the shelter home] that I am a liar; I never married and I got pregnant because I was willing to have sex with the guy*.

The rare contact with social workers in charge and untrustworthy social workers made the girls feel that they cannot rely on them. For these reasons most of the girls kept their feelings to themselves rather than talk about them.

#### 3.2.2 Fear and Discrimination Due to Misuse of Power

Several of the respondents mentioned the misuse of power among the social workers or the staff in the shelter homes. The staff used “silent treatment” as a way of punishment when a girl broke the rules such as having a fight with another girl. It could last for a certain period of time such as a couple of hours or days. The girls also pointed out that some of the staff used derogatory names for them and their babies such as bitch, prostitute, “anak haram” (forbidden child) as well as cursed their families:

*No one dared to complain about her [the warden]. I wish I could ask the “ustazah” [religious teacher] why she [the warden] called us prostitutes. She [the warden] called us prostitutes. Sometime I thought maybe it was just her [the warden] style but she was not supposed to call us stupid or dumb*.

Two of the respondents who gave up their babies for intra-familial adoptions mentioned that they did not get the opportunity to meet the adoptive families nor did they sign the consent letter in front of the commissioner of oaths, which are common procedures for adoption.

Another respondent, who was the only Indian, felt that she was discriminated against, especially when it involved her religious practice:

*My social worker always says that she has no time for me as she is having something else to do. I asked the headmistress to allow me to go to a temple for prayer and she did, but my social worker doesn’t want to escort me there*.

#### 3.2.3 Attention Give Sense of Belonging

Several respondents also mentioned a sense of belonging at the shelter homes. We noticed a strong trend that usually the feeling did not involve the social workers or warden in charge. However, there were also exceptions:

*Here we have Mak [means mother; the social worker] and, Mama [the chef]. I consider them as my own mother. I don’t want to think about my sadness. I feel like it is so good to stay here. It is really good. Even some of the girls asked me “Don’t you long for home?” I don’t want to go home*.

Most of the time, the girls felt more comfortable to have a conversation with someone from the staff, such as the cook, guards or in-training-counsellors. One of the girls felt thankful as the in-training-counsellor taught her to read as she was illiterate. Another participant expressed that she felt comfortable as she had gone through several counselling sessions before she came to the shelter home. The previous counsellor prepared her mentally for the stay in the shelter home. Occasionally, the girls felt a kind of communion, especially during big celebrations, such as Eid, as they received “angpau” (a money gift) or a new dress from the social workers.

### 3.3 Relationship with Other Girls in the Shelter Homes

#### 3.3.1 “I” Do Not Want to Connect with “Them”

Almost all of the respondents described this theme. Although most of the girls could make friends and be close to the other girls in the shelter homes, most of them chose not to share their feelings or problems. Several of them mentioned that the other girls have their own problems, so they chose to keep their own problems and feelings to themselves rather than being a ‘burden’ to others. Some of them also chose not to share as they felt that the other girls were bad and immature, which makes them think that they are better off than the other girls:

*The girls here are useless. Certain things maybe I could share with them but not all. I know this because I have been in another shelter home before. If I want to share my feelings or problems I would choose someone much more mature, such as the social worker*.

The majority of the girls described selective close relationship as an option to share their feelings and problems as well as to share gifts from parental visits. Most of the girls mentioned closeness to a specific girl in the shelter home because they did not want the other girls to know about their personal life, especially concerning their background or about the father of their baby. However the selective close relationship could turn bitter if they felt that they had been treated the same way as some other girls:

*Emm, but when her [the close friend] mother came to visit her, she gave her a box of cookies. She ate it with someone else, not with me. But yes she gave the others a bit and I just got as much as the other girls too. One of the girls, I noticed, will become closer to her when she has food. I told Anje [the close friend] and she said she noticed it too*.

In addition, two of the respondents experienced being assaulted verbally by other girls in the shelter homes. They normally quarrel because of misunderstandings about their personal belongings and food.

#### 3.3.2 Joint Activities Facilitate Mutual Relationship

The girls in the shelter homes undertook several recreational activities together, such as applying henna or playing games and tried to live a normal life. One of the girls said; “We joke and laugh. So it reduces my stress for a while.” Another example of mutual relationship was when the girls helped each other either by sharing the household chores or by comforting each other, especially the newcomers. The girls also maintained the mutual relationship through reminding each other of their misbehaviour. In that way, they helped each other to behave according to the rules in the shelter homes. Some of these reminders included table manners, sleeping positions, dress codes and not being naked while taking a shower together. Some of them advised the other girls in a gentle way but most of them reminded the others in a harsh way and the reminder became offensive:

*Tuty told the head of social workers that I like to caress my private part. I didn’t. I swear to God, I didn’t do that. I rubbed it but I didn’t stroke it. They said I caressed through my panties. When did I do that? And they said I have no shame to do that in front of them. It was really annoying. Then I bashed them back*.

Two of the girls had been selected as leaders by the shelter homes administration, thus this situation put them in a dilemma concerning the mutual relationship. As a leader they had responsibilities to observe the other girls and to inform the administration if any of the girls has problems, especially in relation to the other girls. In some way these responsibilities jeopardized the mutual relationship with the other girls:

*I am a “nakibah” [the house leader]. So the warden normally asks me if the girls are having any problems. But I don’t know about the girls’ problems. Because when they knew that I was the house leader, they ceased to talk to me about any problems they may have*.

## 4. Discussion

The aim of the investigation was to highlight the unwed teenage mothers’ voices but also to problematize the Malaysian authorities’ interventions regarding unwed teenage mothers in light of the Convention on the Rights of the Child.

The unwed teenage mothers received few or hardly any counselling sessions with social workers. The situation made the girls think that their feelings and problems were unimportant. As a result the girls seemed withdrawn and became closer to the lower stratum of staff, such as cooks, guards or in-training counsellors, who have less power in the shelter homes. The girls also stated that they experienced being glared at and called bad names, such as prostitute by the staff. Consequently the girls learnt from their surroundings, and, unintentionally, did the same to the other girls. Some of them saw the other girls as inferior and tried to improve their self-image by looking down on the others and seeing them as worthless. They failed to see their similarities and missed working together to face their difficult situation. Sometimes the unfavourable relationship with the other girls, as well as their emotional insecurity to talk about their feelings and problems, derived from the labelling attitude of the staff. The verbal and non-verbal oppression disgraced these girls, evoking a feeling of shame and ridicule of their potential for positive development. Nathanson (1994) indicated that shame leads to psychological and physical processes, such as being defective, weak, inadequate, and unable to control bodily functions. As emphasized in the Convention on the Rights of the Child; the girls have equal rights and value as any other girl in society (UN General Assembly, 1989). It is important for the professionals to change their social prejudice in order to give emotional security and help the girls to build healthy shame, as safety is the priority for positive adaptation (Bonanno, 2004; Herman, 1992).

All of the shelter homes had strict rules and regulations, especially concerning contact with their social networks outside the shelter homes. Significantly, the majority of the girls spoke about their longing to have contact with their families either through face-to-face visits or by other mediums, such as phone calls or letters. The girls experienced their families as their social support and strength that could empower them. The limitations for the relationship that they yearned for and the available but unfavourable relationships made these girls despair. The girls may feel completely humiliated by their helplessness, which could lead to a vulnerable and undesirable situation (Herman, 1992). The Convention on the Rights of the Child also affirmed that the girls have the right to maintain their personal relationship, especially with their parents while being taken care out of their family (UN General Assembly, 1989). Moreover the girls perceived their family as their source of strength. According to Marshall and Baffour (2011), the young residents in shelter homes are keen to have regular contact with family. The pressure could be overwhelming when the girls are returning to their family if they are unaccepted. Thus, the restrictions on contact with their family imposed by the shelter homes could be seen as contravening the Malaysian national laws and international convention (Care Centre Act, 2006; Child Act, 2006; UN General Assembly, 1989). Research by Robichaud, Durand, Bedard and Ouellet (2006) suggested that the crucial qualities for residents in the shelter homes are being treated with respect, having access to relationships and positive approaches from staff. In addition, the girls also talked about the inadequacies concerning the activities in the shelter homes. In other words the girls received less training than that stated in the Malaysian national laws (Care Centre Act, 2006; Child Act, 2006) and UN international mandate (UN General Assembly, 1989). As the girls also had less or no sessions with social workers or counsellors, it left the girls with unoccupied free time, and, according, to Marshall and Baffour (2011), a lack of socially engaging activities for young residents in the institutionalized environment could lead to severe behavioural or mental health problems.

Indeed, the situation in the shelter homes described by the respondents seems less healthy than that expected by the national laws and international standards to promote psychological recovery and the social reintegration of the girls (UN General Assembly, 1989). To ensure the well-being of teenage mothers, it is compulsory to help them and adhere to these standards of service by the enforcement of Malaysian national laws – Care Centre Act (2006) and Child Act (2006) – as well as implementation of the UN mandate (UN General Assembly, 1989).

### 4.1 Conclusions and Recommendations

Despite the intention of the Malaysian national laws – Care Centre Act (2006) and Child Act (2006) – to adhere to the international standard (UN General Assembly, 1989); this study show that almost all of the respondents experienced inadequacy in supervision, rehabilitation and training. Based on our findings, in order for the shelter homes to achieve their crucial functions in protection, supervision, rehabilitation and training, as well as in promoting health and well-being, we strongly suggest the Malaysian authorities to;

- Provide a clear guideline to accomplish the intention of the Malaysian Care Centre Act (2006) and the Convention on the Rights of the Child (UN General Assembly, 1989) concerning the activities/programmes offered as well as the rules and regulations in the shelter homes. It is suggested that the classes/programmes offered should be more attractive and take physical, mental and spiritual aspects into consideration (Azizi & Abd Wahab, 2008; Azizi et al., 2010; Hamilton & Lobel, 2008). The classes/programmes should include sports, vocational training or short courses that could lead to a profession or job, as well as provide religious, social and health education. The courses should also focus on social-cognitive and social-emotional skills (Knorth, Harder, Zandberg, & Kendrick, 2008). Special classes should be offered to those who are illiterate. The guidelines should also consider the expected duration for the planned intervention in order to have better outcomes for the residents (Linqvist, 2011). Qualifications and/or training of the social workers in the shelter homes are other areas in need of attention.
- Enhance the capacity of shelter home staff by introducing them to the social work ethical codes in order to counteract prejudice towards unwed teenage mothers. The professionals are encouraged to facilitate the girls in altering their self-image to avoid the risk of recidivism, failure at school, or involvement in criminal behaviour (Barnet et al., 2009; Omar et al., 2010). There is a need for the professionals to help the girls through group counselling and team building to make them see their similarities and work together in order to facilitate resilience, rebuild their self-image and maximize their mutual relationship (Bonanno, 2004; Bonanno & Mancini, 2008; Clinton, 2008). In addition the staff may function as role models for the girls by promoting positive behaviour towards the girls not only during their stay in shelter homes but also after they have left the shelter homes (Gallagher & Green, 2012). In addition, it is strongly suggested that the staff inculcate unity between the girls and their family in order to function as a correctional rehabilitation centre as well as to prevent recidivism (Sarnon et al., 2012)
